# The Relationship between Liver Volume, Clinicopathological Characteristics and Survival in Patients Undergoing Resection with Curative Intent for Non-Metastatic Colonic Cancer

**DOI:** 10.3390/tomography10030027

**Published:** 2024-02-28

**Authors:** Josh McGovern, Charles Mackay, Rhiannon Freireich, Allan M. Golder, Ross D. Dolan, Paul G. Horgan, David Holroyd, Nigel B. Jamieson, Donald C. McMillan

**Affiliations:** Academic Unit of Surgery, School of Medicine, University of Glasgow, New Lister Building, Royal Infirmary, Glasgow G31 2ER, UK; 2362176m@student.gla.ac.uk (C.M.); rhiannon.freireich5@icloud.com (R.F.); donald.mcmillan@glasgow.ac.uk (D.C.M.)

**Keywords:** liver volume, cancer, survival

## Abstract

Introduction: The prognostic value of CT-derived liver volume in terms of cancer outcomes is not clear. The aim of the present study was to examine the relationship between liver area on a single axial CT-slice and the total liver volume in patients with colonic cancer. Furthermore, we examine the relationship between liver volume, determined using this novel method, clinicopathological variables and survival. Methods: Consecutive patients who underwent potentially curative surgery for colonic cancer were identified from a prospectively maintained database. Maximal liver area on axial CT-slice (cm^2^) and total volume (cm^3^), were obtained by the manual segmentation of pre-operative CT-images in a PACS viewer. The maximal liver area was normalized for body height^2^ to create the liver index (LI) and values, categorized into tertiles. The primary outcome of interest was overall survival (OS). Relationships between LI and clinico-pathological variables were examined using chi-square analysis and binary logistic regression. The relationship between LI and OS was examined using cox proportional hazard regression. Results: A total of 359 patients were included. A total of 51% (*n* = 182) of patients were male and 73% (*n* = 261) were aged 65 years or older. 81% (*n* = 305) of patients were alive 3-years post-operatively. The median maximal liver area on the axial CT slice was 178.7 (163.7–198.4) cm^2^. The median total liver volume was 1509.13 (857.8–3337.1) cm^3^. Maximal liver area strongly correlated with total liver volume (R^2^ = 0.749). The median LI was 66.8 (62.0–71.6) cm^2^/m^2^. On multivariate analysis, age (*p* < 0.001), sex (*p* < 0.05), BMI (*p* < 0.001) and T2DM (*p* < 0.05) remained significantly associated with LI. On univariate analysis, neither LI (continuous) or LI (tertiles) were significantly associated with OS (*p* = 0.582 and *p* = 0.290, respectively). Conclusions: The simple, reliable method proposed in this study for quantifying liver volume using CT-imaging was found to have an excellent correlation between observers and provided results consistent with the contemporary literature. This method may facilitate the further examination of liver volume in future cancer studies.

## 1. Introduction

CT-derived volumetry facilitated the measurement of liver volume in malignant and non-malignant pathologies [1,2]. Both age and anthropometric measures, such as body mass index (BMI) and body surface area (BSA), were found to be robust determinants of liver volume [3,4]. Furthermore, the pathology of the liver itself, such as non-alcoholic fatty liver disease (NAFLD) and co-morbid diseases including diabetes mellitus, are also thought to affect liver volume [5,6]. In contrast, the effect of cancer on liver volume using CT-derived volumetry has rarely been examined.

While the liver is generally considered to be preserved/increase in size in patients with cancer [7], only a handful of studies to date have examined CT-derived liver volume in patients with cancer [8,9,10]. Furthermore, the observations of contemporary studies may have been confounded by several factors, including the administration of certain chemotherapy agents and the burden of metastatic disease in the liver itself [11,12]. As such, the relationship between liver volume and cancer remains unclear. Similarly, it is unclear whether cancer influences the relationships between liver volume and clinicopathological characteristics. A delineation of these relationships may not only further our understanding of the effect of cancer on the liver and but provide a foundation from which the measurement of the liver may be incorporated in future studies of cancer-associated wasting [13,14].

The paucity of studies examining CT-derived liver volume in patients with cancer is likely attributable to the time-consuming nature of manual segmentation and the absence of a standardised methodology [15,16]. Nevertheless, despite the advances in semi-automated/automated measurement of liver volume for CT-imaging, manual segmentation is still regarded by many as the gold standard [1,2]. Indeed, manual segmentation has been shown to be a reliable measure of liver volume, with cohort studies showing an excellent correlation between CT-derived measurements and the mass of resected specimens in patients undergoing hepatectomy for colorectal liver metastasis [9,10]. and liver transplantation [17].

The aim of the present study was two-fold. Firstly, we aimed to examine if the liver area on a single axial CT-slice was a reliable marker of the total liver volume, analogous to the current gold-standard methodology for quantifying volume and distribution on CT-imaging [18]. Secondly, we aimed to examine the relationships between liver volume and clinicopathological characteristics in patients with a colonic cancer, who did not receive neo-adjuvant chemotherapy and had no evidence of liver metastasis on pre-operative staging CT imaging.

## 2. Methods

### 2.1. Patients

Consecutive patients who underwent elective, potentially curative, right or extended right hemicolectomies for colonic cancer between the 1 March 2008 and 1 April 2018, within NHS Greater Glasgow and Clyde (NHSGGC), were identified from a prospectively maintained database. Patients who had TNM stage I–III disease and recorded pre-operative height and weight for the calculation of BMI, satisfactory CT imaging for a body composition analysis, and pre-operative assessment of the systemic inflammatory within the preceding 3 months of surgery were assessed for inclusion. Exclusion criteria were as follows: those who had undergone neo-adjuvant chemotherapy and patients who had undergone previous hepatic resections. As this was a retrospective analysis of existing clinical data, formal ethical committee review was not required according to National Research Ethics Service guidance. The need for individual patient consent was waived due to the retrospective observational nature of the study.

Routine demographic details, including age, sex, height and weight, were recorded. Age categories were grouped into <64, 65–74 and >74 years. BMI was categorized as <18.5, 18.5–24.99, 25–29.99 and ≥30 kg/m^2^. Body surface area (BSA, m^2^) was calculated using Mosteller’s formula and categorised into tertiles [19]. Patient comorbidity was classified using the American Society of Anaesthesiologists (ASA) grading system [20]. Furthermore, the presence of Type 2 diabetes mellitus (T2DM) and liver disease (non-alcoholic fatty liver disease/cirrhosis) was also recorded.

The primary outcome of interest was overall survival (OS). Vital status was obtained from the included patients’ electronic case records. The date of the last recorded follow-up or last review of electronic case records was 1 December 2022, which served as the censor date.

### 2.2. Measurements of the Maximal Liver Area and Total Liver Volume

**Figure 1:**
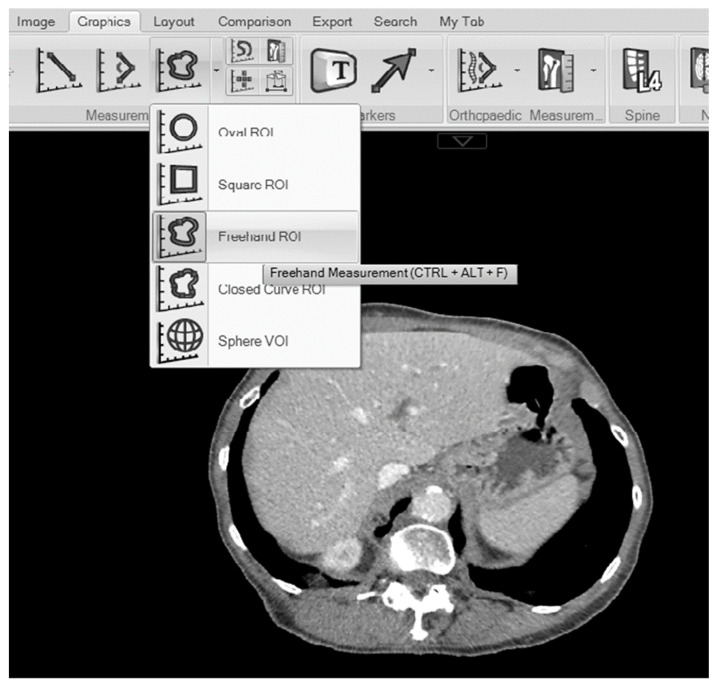
Carestream Vue PACS viewer showing the freehand measurement tool.

Measurements of interest were the maximal liver area on an axial CT slice (cm^2^) and total liver volume (cm^3^). The cross-sectional area of liver (cm^2^) was manually delineated on portal–venous CT scans using the freehand measurement tool available within the Carestream Vue PACS ((Picture Archiving and Communicating System (PACS) Version 12.2) Graphics > measurement > freehand; see Figure 1). The gallbladder and the inferior cava were excluded from the region of interest; however, intrahepatic biliary and vascular structures were included, as previously described in the literature [16]. Where possible, benign liver lesions were also excluded from the region of interest. Measurements were performed by a general surgical specialist registrar (J.M.). A sample of images (*n* = 30) was also analysed by another general surgical specialist registrar (A.M.G.) to examine the inter-rater reliability of the maximal liver area measurement.

Firstly, the maximal axial liver area was identified by the manual delineation of sequential images, which was approximated to be the largest area by the naked eye, from the slice at which the liver first appeared cranially. The median number of slices analysed to identify the CT-slice containing the maximal liver area was 7 (5–9). The maximal liver area on an axial CT slice was then normalized for body height^2^ to create the liver index (LI). Following this, the liver area on sequential axial CT image slices was then manually delineated on all slices, as described above, at 5 mm intervals, from the slice at which the liver first appeared cranially. The sum of all liver area measurements was multiplied by the slice interval (5 mm) to give the total liver volume (cm^3^). A slice interval of 5 mm was selected, as this has been shown to be both time-efficient and provide good correlation with total liver volume in previous studies [15,21].

### 2.3. Statistical Analysis

Correlations amongst maximal cross-sectional liver area on an axial CT slice (cm^2^) and total liver volume (cm^3^) were examined using linear regression and the results showed the coefficient of determination (R^2^). The inter-rater reliability of the maximal cross-sectional liver area on axial CT slice (cm^2^) measurements was examined using inter-class correlation coefficients (ICCCs).

LI tertiles were calculated and patients were grouped into categories according to LI value. The relationship between LI and age, sex, BMI, BSA, ASA, T2DM, liver disease and 3-year survival were examined using the chi-square test for linear-by-linear association. The relationships between LI and clinicopathological characteristics were also examined via uni- and multivariate binary logistics regression analysis. OS was defined as the time (months) from date of surgery to date of death due to any cause. The prognostic value of LI to OS was examined using univariate and multivariate Cox’s proportional hazards model. LI was presented as both a continuous and categorical (tertiles) variable.

Clinicopathological factors that had a *p* value < 0.1 were included in a multivariate model using a backward conditional model to identify independently significant factors. Missing data were excluded from analysis on a variable-by-variable basis. Two-tailed *p* values < 0.05 were considered statistically significant. A statistical analysis was performed using SPSS software version 27.0. (SPSS Inc., Chicago, IL, USA).

## 3. Results

### 3.1. Patient Inclusion

A total of 359 patients met the inclusion criteria. The clinicopathological characteristics of the included patients are shown in Table 1. A total of 51% (*n* = 182) of patients were male and 73% (*n* = 261) were aged 65 years or older. The median BMI of the cohort was 27 kg/m^2^ and 65% (*n* = 234) of patients had a BMI ≥ 25 kg/m^2^. The median BSA was 1.73 m^2^ (1.51–1.94). A total of 39% (*n* = 141) of patients were ASA ≥ 3. A total of 19% (*n* = 69) of patients had T2DM and 4% (*n* = 15) had a documented medical history of liver disease. The median follow-up was 79 (51–109) months. A total of 81% (*n* = 305) of patients were alive 3 years post-operatively (see Table 1).

### 3.2. Relationships between Cross-Sectional Liver Area and Total Liver Volume

The median maximal liver area on axial CT slice was 178.7 (163.7–198.4) cm^2^. The median total liver volume was 1509.13 (857.8–3337.1) cm^3^. The maximal liver area was found to strongly correlate with total liver volume in a randomly selected sample of 50 patients included in the study (R^2^ = 0.749). 

**Figure 2:**
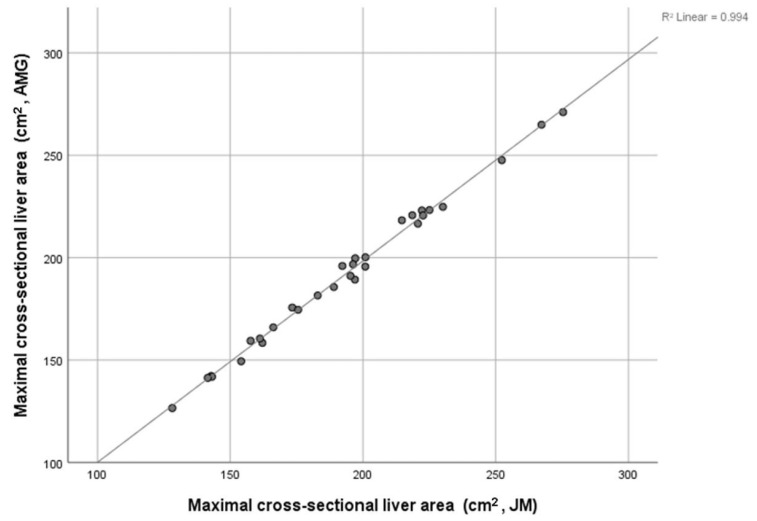
Correlation between maximal liver area measurements (cm^2^) of two observers: J.M. and A.M.G. (*n* = 30, R^2^ = 0.994, *p* < 0.001).

The inter-rater reliability of maximal liver area measurements performed by authors J.M. and A.M.G., in a randomly selected sample of 30 patient images, was assessed using inter-class correlation coefficients (ICCCs). The ICC of the maximal cross-sectional liver area on the axial CT slice was 0.998. The coefficient of determination between measurements showed excellent correlation (R^2^ = 0.994; see Figure 2).

### 3.3. Relationship between Liver Mass Index (LI) and Age, Sex, BMI, BSA, ASA, T2DM, Liver Disease and OS

The median LI was 66.8 (62.0–71.6) cm^2^/m^2^. The relationship LI (tertiles), and age, sex, BMI, BSA, ASA, T2DM, liver disease and OS, are shown in Table 2. Upon univariate analysis, LI was significantly associated with age (*p* < 0.001), BMI (*p* < 0.001), BSA (*p* < 0.001) and T2DM (*p* < 0.001). Upon univariate analysis, LI was not significantly associated with sex (*p* = 0.106), ASA (*p* = 0.053), liver disease (*p* = 0.347) or 3-year survival (*p* = 0.350).

Upon univariate cox regression analysis, neither LI (continuous) or LI (tertiles) were significantly associated with OS (HR 1.00, 95%CI 0.98–1.01, *p* = 0.582 and HR 0.90, 95%CI 0.74–1.09, *p* = 0.290, respectively). Therefore, the results of the survival analysis were not displayed in detail in the manuscript.

### 3.4. Relationship between LI (Lowest/Middle vs. Highest Tertiles) and Age, Sex, BMI, BSA, ASA, ASA and T2DM

The relationship between LI (lowest vs. middle/highest tertiles), and age, sex, BMI, BSA, ASA, ASA and T2DM, is shown in Table 3. Upon univariate analysis, LI was significantly associated with age (*p* < 0.001), sex (*p* < 0.05), BMI (*p* < 0.001), BSA (*p* < 0.001), ASA (*p* < 0.05) and T2DM (*p* < 0.001). Upon multivariate analysis, age (*p* < 0.001), sex (*p* < 0.05), BMI (*p* < 0.001) and T2DM (*p* < 0.05) remained significantly associated with LI.

## 4. Discussion

To our knowledge, the present study is the first to examine the relationship between cross-sectional liver area, obtained by manual segmentation of a single axial CT slice, and total liver volume in patients with cancer. The present results show that there was a strong correlation between maximal liver area and total liver volume using the proposed methodology. Furthermore, the measurement of maximal liver area, obtained utilizing software that is readily available in clinical practice, had excellent inter-rater reliability. Lastly, the present results show that liver volume in patients with non-metastatic colonic cancer was primarily determined by age, sex, BMI and the presence of a co-morbid disease such as T2DM, in keeping with the contemporary non-cancer literature. However, there was no association between liver volume and survival. Therefore, while the prognostic value of liver volume to cancer outcomes is unclear, the present study provides a simple, reliable method to examine liver volume in future studies and is informative regarding the relationship between liver volume and clinicopathological characteristics in patients with cancer.

Manual segmentation of the liver is still regarded by many as the gold-standard methodology for CT–liver volumetrics [1,2]. However, the absence of a standardized methodology and the time-consuming nature of measurement has limited the number of studies examining liver volume in patients with cancer [22,23]. The present results are, therefore, of interest, finding that a single measure obtained by the manual segmentation of axial CT slices, obtained in less than five minutes on pre-existing software that is routinely available in clinical practice, was not only a reliable measure showing an excellent correlation between the measurements taken by independent observers but also had a strong correlation with the total liver volume (see Figure 2). Given that the median total liver volume observed in the present study was comparable with that of other contemporary studies of malignant and non-malignant disease that employed both the manual segmentation of CT images for liver volumetry [1,3,15,24] and semi-automated measures [10,23], the present methodology is likely to be reliable. Further study of other cohorts should readily confirm the present observations and external validity of this novel methodology.

Upon multivariate analysis, liver index (LI) was found to be significantly associated with age, male sex, BMI and T2DM in the present study. The present observations are therefore consistent with those of Vauthey et al., who reported that, in 292 patients from four sites across North America and Europe who underwent CT-imaging for conditions unrelated to the hepatobiliary system and had no known hepatic abnormality, liver volume was correlated with body weight [3]. The results of the present study are also consistent with those of Harada et al., who, in a cohort of 374 consecutive patients who underwent abdominal CT imaging for a range of gastro-intestinal pathologies, found that liver volume was negatively correlated with age [4]. Furthermore, male patients we found to have significantly larger liver volumes than female patients [4]. Lastly, these results are correlated with those of Martin et al., who reported that T2DM was associated with MRI-derived liver volume in 32,859 patients, identified from a UK biobank [5]. Therefore, the present results suggest that the determinants of liver volume in patients with cancer are similar to those of patients without neoplasia. As such, the present observations may provide a basis for future studies of liver volume in patients with cancer, as well as studies on the relationship with clinicopathological characteristics, and studies on other organs, such as skeletal muscle.

There is now a significant volume of literature examining the prognostic value of CT-derived measures of soft tissues for clinical outcomes in patients with cancer [25,26]. In patients with colorectal cancer, both skeletal muscle and fat volume were consistently reported to be associated with survival outcomes [27,28,29]. Automated CT-derived volumetry of other viscera remains an area of interest [30], although the clinical utility of such measures in patients with cancer remains unclear. Indeed, much of the present literature examining the prognostic value of CT-derived liver volumetry was of patients undergoing hepatic resection or liver transplantation [16]. As such, the present results are informative, with no significant association observed between CT-derived liver volume and survival in patients with non-metastatic colonic cancer. However, these results are in no way conclusive given the relatively modest sample size of the present study. Furthermore, they are difficult to compare with the paucity of similar studies examining the prognostic value in patients with colonic cancer. Therefore, further large-cohort studies are required to determine the prognostic value of CT-derived liver volume for clinical outcomes in patients with cancer, analogous to those examining skeletal muscle [31,32] and fat [33].

The liver is thought to be central to the phenotypic alterations in body composition experienced with cancer progression [14,34]. In contrast to soft tissues, the liver is largely considered to be preserved in cancer [7]. However, compared to skeletal muscle and fat, there is a relative paucity of studies utilizing modern-day imaging techniques to examine the alterations in liver mass in patients with cancer. Indeed, the observations from studies utilizing CT volumetry to examine the relationship been liver volume and cancer outcomes are often confounded by several factors, including the administration of certain chemotherapy agents and the burden of metastatic disease in the liver itself [11,12,35]. As such, the present observations are informative, as they show that the relationships between clinicopathological characteristics and liver volume in non-malignant disease are similar in those with CRC. While further study is required to validate the present observations in cancer cohorts not confounded by neo-adjuvant chemotherapy or liver metastasis, the present observations provide a foundation for the incorporation of CT-derived liver volumetry in future studies of cancer-associated wasting [13,14].

The present study has a number of limitations. Firstly, this study was a single-centre study, with a modest sample size, and therefore may be subject to sample bias. Secondly, while a 5 mm slice thickness has been shown to be acceptable for CT liver volumetrics, a smaller slice thickness would reduce error [21]. Nevertheless, while adjusting for tumour site and metastatic disease, the present results are comparable with those of other CT–manual-segmentation studies of liver volume [1,3,15,24]. Thirdly, the absence of a fixed anatomical landmark mark, like the third lumbar vertebra (L3) commonly used in the measurement of soft tissues, is a limitation. Indeed, the correlation between the maximal liver area and total liver volume observed in the present study is not as strong as that reported by studies examining the correlation between single-slice area and multi-slice volumes of muscle/fat at L3 [36]. Nevertheless, the present study shows a strong correlation between single-slice liver area and total liver volume derived using manual segmentation, which is currently considered the gold-standard methodology for liver volumetry [2]. Therefore, this simple and reliable method may facilitate further study of liver volume until semi-automated/automated software for CT-derived volumetry becomes validated and routinely available. Lastly, liver volume was not found to be significantly associated with survival in patients with non-metastatic colonic cancer in the present study. Given the small sample size and good survival outcomes that were observed, further studies with larger cohorts and a range of disease stages are required to determine the prognostic value of liver volume for clinical outcomes in patients with colonic cancer.

## 5. Conclusions

The simple, reliable method proposed in this study for quantifying liver volume using CT imaging was found to have excellent correlation between observers and provide results consistent with the contemporary literature. This method may facilitate the routine measurement of liver volume and allow for an examination of the relationships between liver volume and other organs, particularly skeletal muscle, in patients with cancer.

## Figures and Tables

**Table 1:** Clinicopathological characteristics of included patients (*n* = 359).

	*n* = (%)
Age	
<65	98 (27%)
65–74	128 (36%)
>74	133 (37%)
Sex	
Female	177 (49%)
Male	182 (51%)
BMI (kg/m^2^)	
<18.5	13 (4%)
18.5–24.99	112 (31%)
25–29.99	113 (31%)
≥30	121 (34%)
BSA (m^2^)	
<1.51	119 (33%)
1.51–1.94	120 (33.5%)
>1.94	120 (33.5%)
ASA	
1	53 (15%)
2	165 (46%)
≥3	141 (39%)
T2DM	
No	289 (81%)
Yes	69 (19%)
Liver Disease	
No	341 (96%)
Yes	15 (4%)
Median maximal cross-sectional liver area (cm^2^)	178.7 (163.7–198.4)
Median LI (cm^2^/m^2^)	66.8 (62.0–71.6)
Median total liver volume (cm^3^)	1509.13 (857.8–3337.1)
3-year survival	
Yes	305 (80%)
No	75 (20%)

**Table 2:** The relationship LI (tertiles), and age, sex, BMI, BSA, ASA, T2DM, liver disease and OS (*n* = 359).

	LI < 61.9 cm^2^/m^2^ (*n* = 119)	LI 61.9–71.6 cm^2^/m^2^ (*n* = 120)	LI > 71.6 cm^2^/m^2^ (*n* = 120)	*p* Value ^1^
Age				<0.001
<65	25 (21%)	30 (25%)	43 (36%)	
65–74	36 (30%)	44 (37%)	48 (40%)	
>74	58 (49%)	46 (38%)	29 (24%)	
Sex				0.106
Female	61 (51%)	67 (56%)	71 (59%)	
Male	58 (49%)	53 (44%)	49 (41%)	
BMI (kg/m^2^)				<0.001
<18.5	8 (7%)	3 (3%)	2 (2%)	
18.5–24.99	59 (49%)	40 (33%)	13 (11%)	
25–29.99	31 (26%)	43 (36%)	39 (32%)	
≥30	21 (18%)	34 (28%)	66 (55%)	
BSA (m^2^)				<0.001
<1.51	56 (47%)	38 (32%)	25 (21%)	
1.51–1.94	35 (29%)	46 (38%)	39 (32%)	
>1.94	28 (24%)	36 (30%)	56 (47%)	
ASA				0.053
1	24 (20%)	17 (14%)	12 (10%)	
2	50 (42%)	61 (51%)	54 (45%)	
≥3	45 (38%)	42 (35%)	54 (45%)	
T2DM				<0.001
No	110 (92%)	98 (82%)	81 (67%)	
Yes	9 (8%)	21 (18%)	39 (33%)	
Liver Disease				0.347
No	114 (97%)	114 (97%)	113 (94%)	
Yes	4 (3%)	4 (3%)	7 (6%)	
3-year survival				0.350
Yes	102 (79%)	95 (79%)	108 (83%)	
No	28 (21%)	25 (21%)	22 (17%)	

^1^ *p* value is from chi-square analysis.

**Table 3:** The relationship between LI (lowest/middle vs. highest tertiles) and age, sex, BMI, BSA, ASA, ASA and T2DM (*n* = 359).

	OR (Univariate)	*p*-Value	OR (Multivariate)	*p*-Value
Age (<65/65–74/>74 years)	0.60 (0.45–0.79)	<0.001	0.53 (0.38–0.74)	<0.001
Sex (Female/Male)	1.67 (1.08–2.61)	0.023	2.10 (1.14–3.82)	0.017
BMI (<18.5/18.5–24.9/25–30/>30 kg/m^2^)	2.69 (2.00–3.61)	<0.001	3.04 (1.99–4.65)	<0.001
BSA (<1.51/1.51–1.94/>1.94 m^2^)	1.81 (1.37–2.41)	<0.001	-	0.058
ASA (1/2/≥3)	1.40 (1.01–1.94)	0.043	-	0.058
T2DM (No/Yes)	3.34 (1.94–5.73)	<0.001	2.48 (1.33–4.62)	0.004

Odds ratio, 95%CI, *p* value.

## Data Availability

Data will be made available following request to the senior authors.
